# Retrospective review of bladder biopsy instead of skin biopsy provided clue for the diagnosis of neuronal intranuclear inclusion disease

**DOI:** 10.3389/fnins.2024.1448918

**Published:** 2024-08-12

**Authors:** Jun Liu, Zhenzhu Hu, Weihong Tan, Ying Li, Hao Chen

**Affiliations:** ^1^Department of Neurology, Hospital of Guang’an People’s Hospital, Guang’an, China; ^2^Department of Neurology, Xuzhou Cancer Hospital, Xuzhou, Jiangsu, China; ^3^Department of Clinical College, Chengdu Medical College, Chengdu, Sichuan, China; ^4^Department of Neurology, The Affiliated Hospital of Xuzhou Medical University, Xuzhou, China

**Keywords:** NIID, skin biopsy, bladder biopsy, encephalitic episode and case report, MRI

## Abstract

The purpose of this study is to present a case of a patient with a negative skin biopsy who was diagnosed with neuronal intranuclear inclusion disease (NIID) through a retrospective review of a bladder biopsy specimen obtained 10 years ago. The patient initially presented with encephalitis-like symptoms, including fever, headache, and abnormal mental behavior. However, the DWI hyperintensity in the corticomedullary junction indicated the possibility of NIID. Due to the negative results of the skin biopsy, we initially refrained from hastily diagnosing the patient with NIID. However, 6 months later, the patient was readmitted to the hospital due to similar symptoms, and a follow-up MRI showed significant enlargement of the lesions at the corticomedullary junction. This time we identified intranuclear inclusions in her tissue specimens from bladder surgery. Subsequently, genetic testing was performed, leading to the diagnosis of NIID in the patient. Our case report indicates that detecting intranuclear inclusions from previous surgical specimens, rather than relying solely on skin biopsy, could significantly enhance diagnostic methods for NIID.

## Introduction

Neuronal intranuclear inclusion disease (NIID) is a neurodegenerative disease characterized by the presence of eosinophilic hyaline intranuclear inclusions in the central and peripheral nervous systems as well as in various organs, such as the skin, leading to skin biopsy as an important clue in the diagnosis of this disease (Liu et al., 2022; Bao et al., 2023). However, the sensitivity and specificity of skin biopsy in diagnosing NIID warrant further investigation. For instance, the positive rate of skin biopsy in patients with sporadic NIID can be as high as 97.4%, whereas for familial NIID, it may be as low as 78.9% (Sone et al., 2016). Recent studies suggest that the specificity of skin biopsy for diagnosing NIID might be overestimated, given that intranuclear inclusions are also observed in other conditions associated with GGC repeat expansions, such as fragile X-associated tremor/ataxia syndrome (FXTAS) and oculopharyngodistal myopathy (OPDM) (Ishiura et al., 2019; Toko et al., 2021). Additionally, as an invasive procedure, skin biopsy may not be feasible for all patients. In this study, we present a case of NIID who initially presented with encephalitic episodes but had a negative skin biopsy for intranuclear inclusions. Upon reanalyzing her bladder tissue from a decade ago, we observed the presence of intranuclear inclusions within the bladder tissue.

## Case report

A 63-year-old woman presented to the emergency department of the Affiliated Hospital of Xuzhou Medical University, with a chief complaint of sudden onset of fever (maximum 39.3°C), headaches, accompanied by irritability, anxiety, and incoherence. In the past decade, the individual was diagnosed with bladder neck stenosis and received a bladder neck incision in our hospital. The family history was positive for dementia symptoms in her father and her older sister.

**Figure 1:**
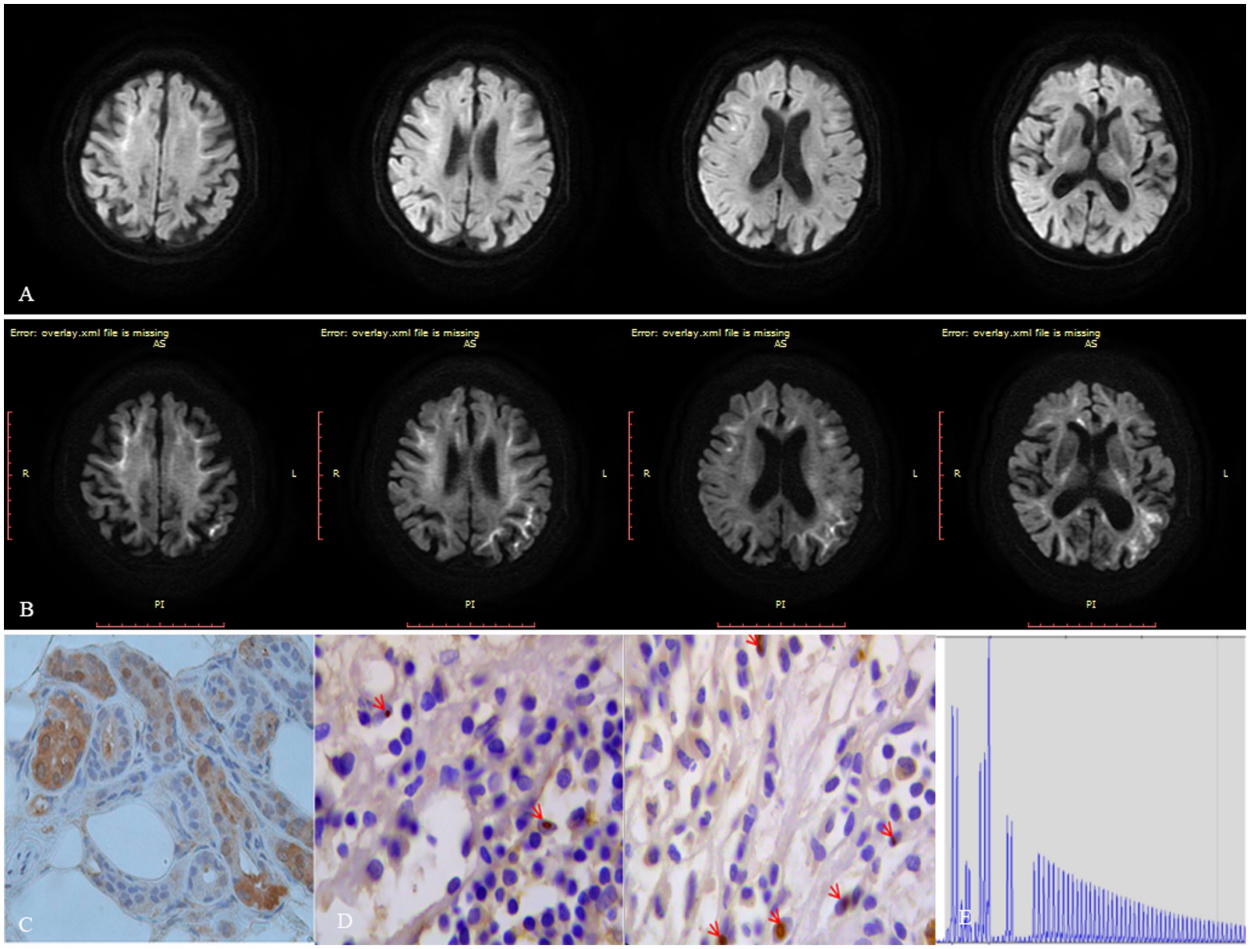
Figure imaging, histopathologic, and genetic analyses of the patient. **(A)** DWI hyperintensity along the corticomedullary junction is limited to the frontal lobe during the first encephalitic episode. **(B)** DWI hyperintensity along the corticomedullary junction extends from the frontal lobe posteriorly to the temporo-parietal-occipital lobe 6 months after the initial encephalitic episode. **(C)** Neither ubiquitin nor p62-positive intranuclear inclusions were detected in her skin tissue. **(D)** Both ubiquitin or p62 positive intranuclear inclusions were detected in her bladder tissue specimen taken 10 years ago. **(E)** Repeat-primed PCR showed 140 GGC repeats in the *NOTCH2NLC* gene in this patient.

On neurological examination, the patient was confused and revealed neck stiffness and positive Kernig’s sign without any other focal neurologic deficits. A cerebrospinal fluid (CSF) examination showed pleocytosis (22 cells) with mononuclear predominance (83.40%) and elevated proteins (1.10 g/L). The microbiological study and autoimmune encephalitis testing of blood and CSF were both unremarkable. Herpes simplex encephalitis was initially suspected, and acyclovir was administrated to this patient without any improvements. Subsequently, the DWI sequence showed high signals along the corticomedullary junction, localized within the bilateral frontal lobes (Figure 1A). We considered that the patient was likely suffering from NIID, with encephalitic episodes as the primary presenting symptom. A skin biopsy was conducted on this patient without detecting the presence of p62 or ubiquitin-positive intranuclear inclusions (Figure 1C). Moreover, the patient refused to undergo genetic testing for detecting GGC expansion in the *NOTCH2NLC* gene, leading to an inconclusive diagnosis of NIID. Fortunately, the patient experienced spontaneous remission of symptoms and requested to be discharged from the hospital. After a 6-month interval, the patient had a relapse of symptoms, including fever, headache, and abnormal mental and behavioral manifestations, as well as a generalized tonic–clonic seizure. A follow-up MRI revealed an expansion of the DWI hyperintensity along the corticomedullary junction, extending posteriorly to the temporo-parietal-occipital lobe (Figure 1B). The patient declined to undergo another skin biopsy, however, we further retrospectively reviewed her bladder specimen 10 years ago and noticed p62 and ubiquitin-positive intranuclear inclusions in her bladder tissue (Figure 1D). Furthermore, genetic testing was performed using Repeat-primed PCR (RP-PCR) and GC-rich PCR (GC-PCR) assays, which confirmed that the patient carried 117 GGC repeats within the NOTCH2NLC gene (Figure 1E), leading to a conclusive diagnosis of NIID. At the 18-month follow-up, the patient suffered dementia symptoms primarily related to frontal lobe dysfunction, such as lack of concentration, memory impairment, and behavioral changes.

## Discussion

The clinical presentation of NIID is characterized by a wide range of neurological symptoms that can closely resemble those of other neurodegenerative disorders, such as Alzheimer’s disease (AD), essential tremor (ET), Parkinson’s disease (PD), amyotrophic lateral sclerosis (ALS), and oculopharyngodistal myopathy (OPDM), rendering its clinical diagnosis a complex and challenging task (Liu et al., 2022; Bao et al., 2023). Despite being challenging to diagnose, NIID can be distinguished from other neurodegenerative conditions by the presence of paroxysmal symptoms in affected patients, including disturbances in consciousness, stroke-like or encephalitic episodes, generalized convulsions, and migraine-like symptoms (Tian et al., 2022; Tai et al., 2023). A recent multicenter retrospective study has revealed that paroxysmal symptoms are the most prevalent clinical manifestation of NIID, with approximately 66.8% of affected patients suffering such symptoms at some point during the course of the disease (Tian et al., 2022). In our case, the patient did not have a previous medical history of neurological diseases, and the presence of sudden onset fever, headache, and unusual mental behavior led to the initial diagnosis of viral encephalitis. However, DWI hyperintensity along the corticomedullary junction in this patient strongly suggested that her initial encephalitic episode was the first manifestation of NIID. Notably, according to previous reports, approximately 32.8% of NIID patients may experience paroxysmal symptoms during the early stages of the disease (Tian et al., 2022).

The absence of inclusions in the patient’s skin tissue led us to suspect the initial diagnosis of NIID. The identification of p62 and ubiquitin-positive intranuclear inclusions through skin biopsy has long been recognized as a hallmark pathological feature of NIID and was previously considered a prerequisite for the diagnosis of NIID before the causative gene was identified (Sone et al., 2011). According to a previous report, the positivity rate for skin biopsies in sporadic NIID patients is as high as 97.4%, however, in familial cases of NIID, the positivity rate is relatively lower at 78.9% (Sone et al., 2016). Therefore, a negative skin biopsy cannot be used as an exclusion criterion for NIID. In a prior publication, we documented the detection of intranuclear inclusions in surgical specimens obtained from various tissues of 24 NIID patients (Chen et al., 2020). Given the negative result of the patient’s skin biopsy, we decided to investigate the presence of intranuclear inclusions in her previous bladder tissue specimen. Fortunately, we were ultimately able to confirm the existence of intranuclear inclusions that were positive for p62 and ubiquitination in the patient’s bladder tissue.

The negative skin biopsy result for this patient may be attributed to several factors. NIID is a highly heterogeneous disease affecting multiple systems, and pathological characteristics in skin tissues can vary among different patients (Chen et al., 2020). Therefore, some individuals may not exhibit the typical intranuclear inclusions, resulting in negative biopsy findings. Additionally, the accuracy of biopsy results can be influenced by the sampling site; if the biopsy sample is not taken from the lesion-affected area, it may fail to capture the inclusions. Furthermore, the patient’s familial history is significant, as both the father and sister have clinical manifestations of NIID, indicating a familial form of the disease. Studies have indicated that skin biopsy positivity rates in familial NIID cases are lower, around 78.9%, which could also contribute to the negative biopsy result observed in our patient (Sone et al., 2016).

Recently, there have been significant advancements in the pathological diagnosis of NIID. Our research team has discovered that characteristic intranuclear inclusions can also be identified through lip gland biopsy and peripheral blood mononuclear cells (PBMCs). These methods offer several advantages: they are easier to obtain, less invasive, and fewer postoperative complications (Bao et al., 2024a,b). Our present case report introduces a novel approach to the pathological diagnosis of NIID: re-evaluating previous surgical specimens to determine the presence or absence of eosinophilic intranuclear inclusions.

## Data availability statement

The original data are available from the first author upon reasonable request.

## Ethics statement

The studies involving humans were approved by Ethics Committee of Xuzhou Medical University within which the work was undertaken has approved the research project. The studies were conducted in accordance with the local legislation and institutional requirements. The participants provided their written informed consent to participate in this study. Written informed consent was obtained from the individual(s) for the publication of any potentially identifiable images or data included in this article.

## Author contributions

JL: Writing – review & editing, Writing – original draft, Methodology, Formal analysis. ZH: Writing – review & editing, Writing – original draft, Formal analysis. WT: Formal analysis, Writing – original draft. YL: Writing – original draft. HC: Writing – review & editing, Visualization, Supervision, Resources, Investigation, Formal analysis.
